# The Cumberland Infirmary, Carlisle

**Published:** 1893-02-25

**Authors:** 


### The Institutional Workshop.

### WITHIN THE HOSPITALS.

### THE CUMBERLAND INFIRMARY, CARLISLE.

(BY OUR SPECIAL COMMISSIONER.)

The buildings of this institution are pleasantly situated in the highest part of the town. They consist of an old central wing erected rather more than fifty years ago, to which has been added during recent years a pavilion of two storeys containing four excellent wards. The older portions of the building contain a number of wards, and the officers' quarters, kitchen, board-room, and other offices. The new pavilion is divided in the centre by a central staircase of handsome proportions, to which a ward opens out on each side. These wards are well proportioned, and contain abundance of air, light, cubic and superficial space. The baths and lavatories are adjacent to the staircase on either side. They are kept in a state of most perfect order, and contain all the modern appliances with the exception of a slop sink. Those at present provided cannot be used for this purpose, but should be replaced as early as possible by the substitution of the slop sinks now used at the Royal Infirmary, Liverpool. As it is, much additional work is thrown upon the nursing staff, which is in our opinion too small for the work to be done, seeing that the beds are generally in constant occupation. There is a small out-patient wing which presents no special features. The mortuary is a surprisingly bad one, but, as it is about to be replaced by a new building, we need not specially refer to it. The kitchen is situated in the basement, a very inconvenient arrangement, which, we fear, cannot now be altered. The sleeping accommodation for the servants is also in the basement, and as so large a portion of the rooms occupied are below the ground level, we are surprised it is possible for them to be used for the purpose to which they are put without serious injury to the health of the domestic staff. No time should be lost in making other and better arrangements for their accommodation. There is evidence that the medical and surgical practice at this hospital is excellent, but the abundant use of iodiform makes the atmosphere of the wards particularly unpleasant.

NURSING DEPARTMENT.

The nursing department made great improvement under the late Matron, Miss Allan. Miss Hamilton, the present Matron, an old St. Thomas's Sister, is fully up to her work. We congratulate the committee on her appointment. The sum of £2,000 has been raised to build a nurses' home as a memorial to the late Bishop of Carlisle. We hope that in settling the plans for this home the Committee will have the wisdom to provide sufficient accommodation, and not to confine it within narrow limits. At the present time the sleeping accommodation for nurses and probationers, and the absence of a proper sitting-room, makes it unusually difficult for this department of the hospital. We would advise them to provide a separate kitchen in the new home, so as to secure full attention to the comfort of the nursing staff, an important matter too often overlooked. We observe that at the present time paying probationers are admitted to this hospital on payment for twelve months' training, and, if satisfactory, a twelve months' certificate of training is given. We should be glad to hear that the Committee had determined not to take probationers, whether paying or working, except for a less period than three years, as it is undesirable to encourage anyone to gain an imperfect knowledge of nursing, especially for a monetary consideration. We would further suggest that instead of binding probationers to give three months and pay a forfeit of £20 should they leave the infirmary before the expiration of their three years' training, that probationers should receive, say £10 a year during the first year, £5 of which should be retained by the hospital to be paid to them at the expiration of three years, or to be forfeited should they leave the service earlier. There is a home-like, comfortable air about the whole institution; the resident staff are most devoted and zealous; and we congratulate the Committee that most things connected with the Cumberland Infirmary are marked by efficiency of arrangement. With the exception of the absence of accommodation, to which we have referred, this institution is one which the managers and staff have a right to be proud of.

